# Distinct and common subcortical functional connectivity revealed across three major psychiatric disorders

**DOI:** 10.1017/S0033291726103377

**Published:** 2026-04-28

**Authors:** Wan-Chen Chang, Duan-Pei Hung, Mu-Hong Chen, Tung-Ping Su, Ya-Mei Bai, You-Yin Chen, Pei-Chi Tu

**Affiliations:** 1 Department of Biomedical Engineering, National Yang Ming Chiao Tung University, Taipei, Taiwan; 2 Department of Medical Research, Taipei Veterans General Hospital, Taipei, Taiwan; 3 Department of Psychiatry, Taipei Veterans General Hospital, Taipei, Taiwan; 4 Department of Psychiatry, Faculty of Medicine, National Yang Ming Chiao Tung University, Taipei, Taiwan; 5 Department of Psychiatry, Cheng Hsin General Hospital, Taipei, Taiwan; 6 Institute of Brain Science, National Yang Ming Chiao Tung University, Taipei, Taiwan; 7 The Ph.D. Program for Neural Regenerative Medicine, College of Medical Science and Technology, Taipei Medical University, Taipei, Taiwan; 8 Institute of Philosophy of Mind and Cognition, National Yang Ming Chiao Tung University, Taipei, Taiwan

**Keywords:** bipolar disorder, functional connectivity, major depressive disorder, schizophrenia, subcortical

## Abstract

**Background:**

Subcortical nuclei – including the thalamus, basal ganglia, and hippocampus-amygdala complex – are key regions in schizophrenia (SZ), bipolar disorder (BD), and major depressive disorder (MDD). While cortical–subcortical connectivity is well studied, fine intra- and inter-subcortical patterns are less known. This study aimed to identify shared and distinct functional connectivity alterations across SZ, BD, and MDD using a high-resolution subcortical atlas.

**Methods:**

Resting-state functional magnetic resonance imaging data from 800 participants (200 per group: SZ, BD, MDD, and healthy controls) in a single-site cohort were analyzed. Subcortical structures were parcellated into 27 regions per hemisphere – thalamus (8 regions), hippocampus (5 regions), amygdala (2 regions), and striatum (12 regions) – based on the a priori atlas. Pairwise functional connectivity among the 54 regions was computed for each participant, and group differences were assessed using general linear models.

**Results:**

Patients with SZ exhibited significantly reduced intra-thalamic connectivity and increased intra-striatal connectivity, as well as enhanced connectivity between the thalamus, striatum, and limbic regions. Patients with BD showed reduced intra-thalamic and intra-striatal connectivity, along with decreased thalamus–amygdala and thalamus–striatum connectivity. In MDD, the predominant finding was reduced intra-limbic connectivity, accompanied by mild reductions in intra-thalamic and striatum–limbic connectivity.

**Conclusion:**

The results suggest that intra-thalamic hypoconnectivity appears common to SZ, BD, and MDD, with graded degrees of severity. In contrast, distinct alterations in intra-striatal and striatum–limbic connectivity may differentiate mood disorders from SZ. These shared and disorder-specific subcortical connectivity patterns enhance the understanding of psychiatric neurobiology and may guide the development of targeted, disorder-tailored interventions.

## Introduction

Schizophrenia (SZ), bipolar disorder (BD), and major depressive disorder (MDD) are among the most prevalent and debilitating psychiatric conditions (Kennedy et al., 2014; Otte et al., 2016; Sajatovic, 2005; Zhong et al., 2024). Despite advancements in pharmacological interventions, neuromodulation options, and improving psychosocial support systems, mental health providers continue to face challenges such as treatment resistance and suboptimal outcomes (Fountoulakis et al., 2020; Howes et al., 2009; Kane, 2012; Voineskos, Daskalakis, & Blumberger, 2020). This is partly due to the remaining obscurity of disease mechanisms, although significant insights have been gained from research over the past years (Chuhma et al., 2017).

Functional connectivity (FC) analysis using resting-state functional magnetic resonance imaging (fMRI) has emerged as a pivotal tool for identifying network abnormalities in major psychiatric disorders. In patients with SZ, converging evidence indicates hypoconnectivity between frontoparietal regions and the striatum, as well as between thalamocortical and cerebellar circuits, alongside hyperconnectivity with sensorimotor areas within the cortical-striatal-thalamo-cortical loops (Voineskos et al., 2024). Research on BD has consistently demonstrated reduced top-down FC from the prefrontal cortex to the amygdala, which is proposed as a trait marker across various mood states (Martino & Magioncalda, 2022; Vargas, Lopez-Jaramillo, & Vieta, 2013). Similar patterns have been reported in patients with MDD, including hyperconnectivity within the default mode network, abnormal activation of the salience network, decreased activation of the executive control network, heightened amygdala activity, and reduced activation of the ventral striatum (Greicius et al., 2007; Hamilton et al., 2018). Notably, there are shared thalamo-cortical alterations of FC across SZ, BD, and MDD (Tu et al., 2020). However, direct comparisons of fMRI connectivity patterns among SZ, BD, and MDD remain limited.

Being centrally located within the brain and evolutionarily conserved, subcortical regions such as the thalamus and basal ganglia function as critical hubs for the integration of motor, sensory, cognitive, and emotional processes. While prior connectivity research has primarily emphasized abnormalities in cortical–subcortical interactions, emerging evidence suggests that FC within subcortical structures themselves may also constitute an important, although underexplored, feature. For instance, recent studies have reported reductions in FC among distinct thalamic subregions – a phenomenon referred to in this study as intra-thalamic connectivity – in individuals with SZ. These deficits are supported by findings indicating that the thalamus frequently emerges as a top-ranking region in whole-brain seed-based FC analyses using the entire thalamus as a region of interest (ROI). Several studies have documented reduced intra-thalamic FC (Anticevic et al., 2014; Ferri et al., 2018; Fryer et al., 2022; Gong et al., 2019), and a meta-analysis has further corroborated these findings, identifying a significant cluster of thalamic hypoconnectivity within the right thalamus (Ramsay, 2019). Moreover, intra-thalamic FC deficits have also been observed in patients with treatment-resistant depression (Tu et al., 2025), suggesting this may represent a transdiagnostic feature across psychiatric disorders.

A recent data-driven approach developed by Tian, Margulies, Breakspear, and Zalesky (2020) has enhanced the parcellation of subcortical regions, offering a refined framework for evaluating FC both within and between these structures. Additionally, the utilization of a large-scale, single-site dataset reduces methodological variability associated with differences in analytical techniques and scanner hardware across studies. This study aims to enhance our understanding of subcortical FC in major psychiatric disorders by examining detailed connectivity patterns within and between key subcortical regions. Subcortical structures such as the thalamus and basal ganglia often serve as relay stations, with their connectivity acting as a proxy for cortical communication. We focused on intra- and inter-subcortical connectivity, informed by neurobiological and statistical reasoning. While traditionally seen as relay stations, recent evidence shows that these regions also function as integrative hubs that modulate communication between large cortical networks (Bell & Shine, 2016; Ho et al., 2022; Hwang, Bertolero, Liu, & D’Esposito, 2017; Kumar, Beckmann, Scheffler, & Grodd, 2022; Tu et al., 2020). By isolating these dynamics, we characterized the ‘gating’ mechanisms that precede broader cortical involvement.

## Methods

### Participants

The study included 800 participants (200 each with SZ, BD, MDD, and healthy controls [HCs]) recruited from both the outpatient and inpatient services of Taipei Veterans General Hospital, a single site in Taiwan. Diagnoses were made after clinical interviews and Mini International Neuropsychiatric Inventory Plus (MINI), according to Diagnostic and Statistical Manual of Mental Disorders, Fourth Edition. Exclusion criteria were as follows: (1) substance abuse or dependence issues in the past 6 months; (2) head trauma with sustained loss of consciousness and/or with cognitive sequelae; and (3) neurological diseases or conditions that affects cerebral metabolism. HC were enrolled from advertisements, who received interviewed by an experienced psychiatrist to exclude people with major psychiatric illness using MINI. People whose first-degree relatives had Axis-I disorders such as SZ, BD, and MDD, were excluded from HC. We assessed clinical symptoms using Positive and Negative Syndrome Scale (PANSS), Hamilton Depression Rating Scale, and Montgomery–Åsberg Depression Rating Scale (MADRS). Before the study, the patient groups had received treatment with a variety of atypical antipsychotics, antidepressants, and mood stabilizers (Supplementary Table 1). All study procedures were reviewed and approved by the Institutional Review Board of Taipei Veterans General Hospital, adhering to the principles outlined in the Declaration of Helsinki. Written informed consent was obtained from all participants following a detailed explanation of the experimental protocols.

### MRI acquisition

MRIs were acquired using a 3.0 Tesla GE Discovery 750 whole-body high-speed imaging device with an eight-channel high-resolution brain coil. Head stabilization was achieved with cushioning, and all the participants wore earplugs (29 dB rating) to attenuate the noise. Automated shimming procedures were performed, and scout images are obtained. The resting-state functional images are collected using a gradient echo T2* weighted sequence (TR/TE/Flip = 2500 ms/30 ms/90°). Forty-three contiguous horizontal slices parallel to the inter-commissural plane (voxel size: 3.5 × 3.5 × 3.5 mm) were acquired and interleaved. These slices cover the cerebellum for each participant. During the functional scans, participants were instructed to remain awake with their eyes open (each scan lasted 8 min and 24 s across 200 time points). In addition, a high-resolution structural image is acquired in the sagittal plane using a high-resolution sequence (repetition time [TR] = 12.54 ms, echo time [TE] = 5.18 ms, inversion time [TI] = 450 ms, flip angle = 12°) and an isotropic 1 mm voxel (FOV 256 × 256).

### FC preprocessing

All preprocessing was performed using the Data Processing Assistant for Resting-State fMRI (http://www.restfmri.net), which is based on Statistical Parametric Mapping (http://www.fil.ion.ucl.ac.uk/spm) and the Resting-State fMRI Data Analysis Toolkit (http://www.restfmri.net). The functional scans received slice-timing correction and motion correction and were normalized to a standard anatomical space (Montreal Neurological Institute). To prepare the data for FC analysis, the following additional preprocessing steps were used: (1) spatial smoothing by using a Gaussian kernel (6-mm full width at half-maximum), (2) temporal filtering (0.009 Hz < f < 0.08 Hz), and (3) removal of spurious or nonspecific sources of variance through the regression of the following variables: (a) Six head motion parameters and autoregressive models of motion: 6 head motion parameters, 6 head motion parameters one time point before, and the 12 corresponding squared items (Friston 24-parameter model); (b) the mean whole-brain signal; (c) the mean signal within the lateral ventricles; and (d) the mean signal within a white matter mask. Furthermore, the regressors used in the method of scrubbing within regression were included to minimize the effect of head motion on the FC measurement. The regression of each of these signals was computed simultaneously, and the residual time course was then retained for the correlation analysis. We adopted the parcellation of subcortical nuclei from Tian et al. (2020), including the thalamus (8 regions), striatum (12 regions), and hippocampus/amygdala (7 regions) (detailed in Supplementary Figure S1). Pairwise FC among the 54 subcortical ROIs was computed by extracting the mean blood-oxygen-level-dependent signal time series and calculating Pearson correlation coefficients between every pair of ROIs for each participant. Correlation coefficients were Fisher-z-transformed prior to group comparisons.

### Statistical analysis

Group comparisons of demographic data and cognitive performance were conducted using one-way analysis of variance using IBM SPSS statistics version 20 (IBM Corp., Armonk, NY, USA). For FC analysis, in total 4293 pairwise FC comparisons (54 × 53/2 connections × 3 diagnostic groups) were performed, and then used a general linear model to compare connectivity patterns between patient groups and HCs, incorporating relevant demographic variables as covariates of no interest to control for potential confounding factors such as age, sex, and education level. Primary group differences were assessed using independent t-tests on the Fisher-z-transformed coefficients, with the resulting p-values corrected via the Benjamini–Hochberg procedure (Storey, Taylor, & Siegmund, 2003) to control the false discovery rate (FDR) at *q* < 0.05. The effect sizes (Cohen’s d) of each patient group as compared with HC were also estimated using the t-statistic adjusted for age, sex from the independent variable of diagnosis (Nakagawa & Cuthill, 2007) to ensure statistical interpretability and clinical comparability across all pairwise connections.

We conducted a sensitivity analysis using a patient subsample to specifically test the influence of antipsychotic medication status, as these are the most potent and widely used drugs in our patient cohorts with known subcortical effects on the observed connectivity differences. Additionally, we performed an exploratory analysis examining the correlations between intra-subcortical connectivity metrics and primary clinical symptom scores across all three patient cohorts (*p* < 0.05).

## Results

Demographic and clinical characteristics of the sample are summarized in Table 1. The MDD group differed significantly from the other groups in sex distribution, age, and illness duration. Participants with MDD were predominantly female and older on average (38 years) compared to those with SZ (34 years), BD (35 years), and HC (22 years). They also had a shorter illness duration (7 years), relative to 11 years in both the SZ and BD groups. HC participants had slightly more years of education (mean = 15) than those in the SZ and BD groups (mean = 13–14). Clinically, the SZ group had the highest average PANSS score (65), followed by BD (41) and MDD (45). As expected, depressive symptom severity, as measured by the HAMD-17 and MADRS, was highest in the MDD group. Rates of medication use across diagnostic groups are presented by class: antipsychotics, antidepressants, and mood stabilizers.

**Table 1.** The enrolled participants’ demographic data

	SZ	BD	MDD	HC	*F*/*X*^2^	*p*
	*n* = 200	*n* = 200	*n* = 200	*n* = 200		
Sex (M/F)	102/98	96/104	62/138	100/100	21.49	<0.001
Age (years)	34.11 ± 9.86	35.14 ± 11.09	38.02 ± 12.64	33.63 ± 9.28	6.64	<0.001
Education (years)	13.49 ± 2.76	14.32 ± 2.74	13.29 ± 2.90	15.69 ± 1.81	35.12	<0.001
age at onset	22.88 ± 6.86	23.59 ± 9.01	31.27 ± 12.53	37.00 ± 0.00	30.68	<0.001
Duration of illness	11.19 ± 9.05	11.31 ± 8.39	6.91 ± 6.99		12.76	<0.001
PANSS total	65.55 ± 16.67	41.51 ± 9.56	45.08 ± 10.02		164.30	<0.001
Positive subscale	14.97 ± 5.04	8.06 ± 2.20	7.63 ± 1.34		218.64	<0.001
Negative subscale	17.19 ± 5.37	8.90 ± 3.20	8.92 ± 3.30		209.23	<0.001
Psychopathology	33.39 ± 8.67	24.55 ± 6.75	28.29 ± 7.11		55.63	<0.001
HAMD17	7.98 ± 5.16	8.76 ± 6.31	16.29 ± 8.26		83.58	<0.001
MADRS	16.43 ± 6.28	11.72 ± 9.27	23.99 ± 10.67		57.40	<0.001
Medication (%)						
antipsychotics	179 (89.5%)	140 (70.0%)	43 (21.5%)			
antidepressant	58 (29.0%)	65 (32.5%)	130 (65.0%)			
mood stabilizers	128 (64.0%)	90 (45.0%)	90 (45.0%)			

Abbreviations: PANSS, Positive and Negative Syndrome Scale for Schizophrenia; HAMD17, 17-item Hamilton Depression Scales; MADRS, Montgomery–Åsberg Depression Rating Scale; WM, Working memory.

### FC difference within subcortical structures

**Figure 1.**
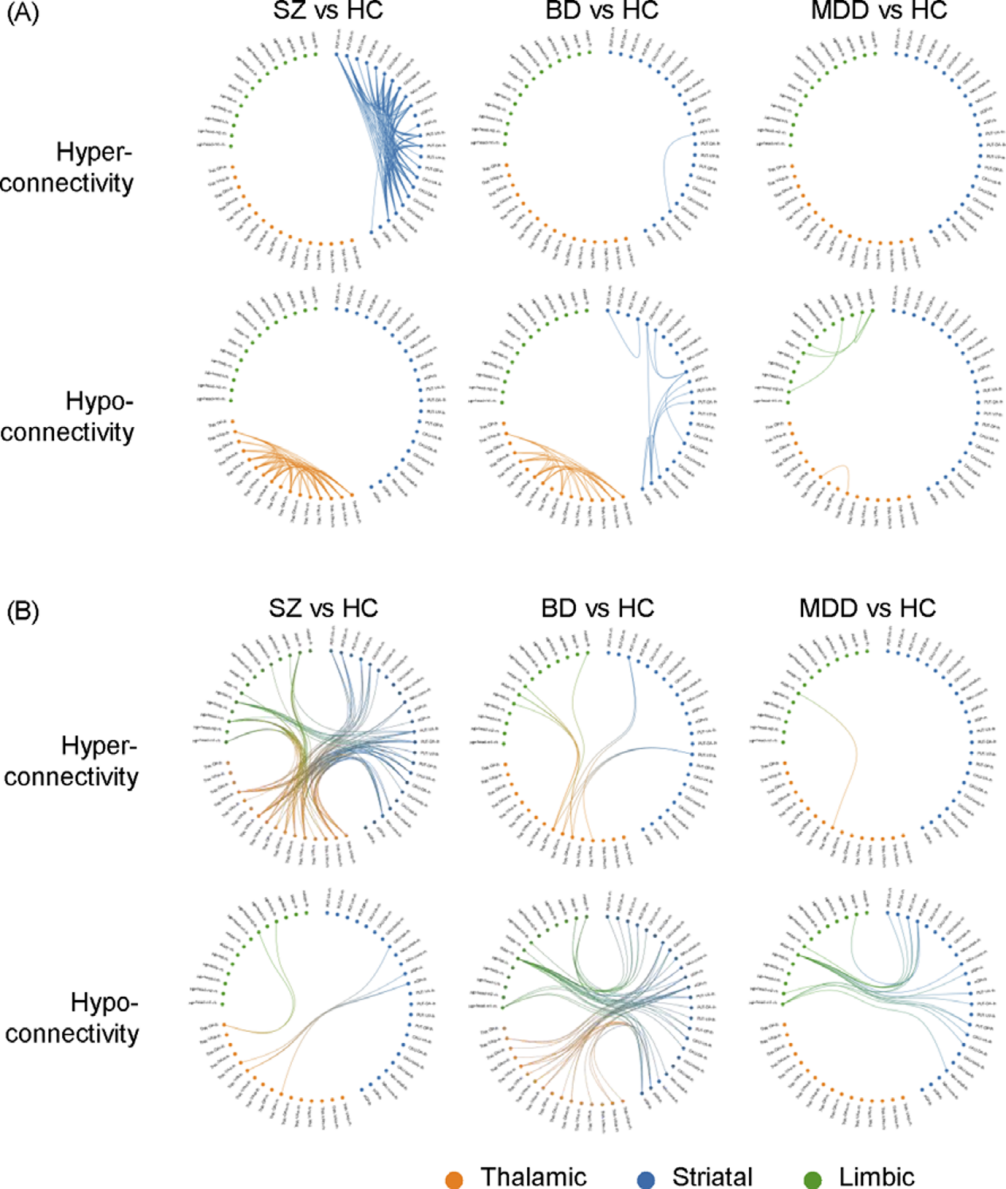
Graphical representation of subcortical functional dysconnectivity in schizophrenia, bipolar disorder, and major depressive disorder relative to healthy controls. The circular subcortical functional connectivity graph displays 52 subregions, categorized into the basal ganglia (blue), thalamus (orange), and hippocampal–amygdala complex (green). (A) Dysconnectivity patterns within each of the three subcortical structures. (B) Dysconnectivity patterns between the three subcortical structures. rh: right hemisphere, lh: left hemisphere.

Group differences relative to controls in FC are presented in Figure 1 and Table 2. Regarding FC abnormalities within individual subcortical structures, patients with SZ exhibited pronounced intra-thalamic hypoconnectivity alongside intra-striatal hyperconnectivity. In contrast, patients with BD were characterized by intra-striatal hypoconnectivity and also demonstrated intra-thalamic hypoconnectivity, albeit to a lesser extent than those with SZ. Patients with MDD displayed prominent intra-limbic hypoconnectivity, including reduced FC between the left medial and lateral amygdala (*p* = 0.0024, *t* = −3.0), between the left medial amygdala and the right lateral amygdala (*p* = 0.0032, *t* = −2.96), and between the left and right hippocampus (*p* = 0.0035, *t* = −2.93) (Figure 1). Additionally, mild intra-thalamic hypoconnectivity was observed in the MDD group, with only one FC reaching statistical significance (*p* = 0.0026, *t* = −3.0). In summary, reduced intra-thalamic FC emerged as a common feature across all three psychiatric disorders. SZ and BD were distinguished by increased and decreased intra-striatal FC, respectively. Finally, decreased intra-limbic FC was identified as a characteristic feature of patients with MDD.

**Table 2.** The ROI pairwise comparisons of functional connectivity between major psychiatric patients and health controls (FDR < 0.05)

ROI54 (total pairs = 1431)	*n* (peakT)		
diff. with HC	SZ	BD	MDD
Decrease			
Intra-thalamic	54 (−7.3)	29 (−5.2)	1 (−3.0)
Intra-striatal	0	11 (−4.2)	0
Intra-limbic	0	0	4 (−3.1)
Inter-B + T	4 (−3.4)	24 (−5.1)	0
Inter-B + A	0	27 (−4.1)	17 (−4.4)
Inter-T + A	2 (−3.0)	0	0
Increase			
Intra-thalamic	0	0	0
Intra-striatal	104 (5.8)	1 (3.0)	0
Intra-limbic	0	0	0
Inter-B + T	53 (5.2)	5 (3.9)	0
Inter-B + A	9 (4.2)	0	0
Inter-T + A	21 (4.8)	5 (4.5)	1 (3.0)
Abnormality (I + D/total pairs, %)	**17.3%**	**7.1%**	**1.6%**

Abbreviations: B, Basal ganglia; T, Thalamus; A, Hippocampal–amygdala; I, Increase; D, Increase.

**Figure 2.**
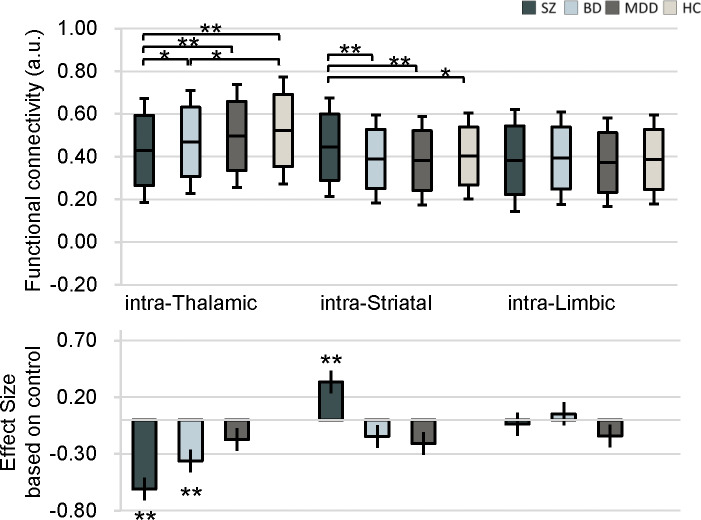
Average intra-nucleus functional connectivity values in patients with schizophrenia, bipolar disorder, and major depressive disorder, as well as healthy controls. Bar plots depict group means, and the accompanying effect sizes represent the magnitude of between-group differences comparing each patient group to healthy controls.

The average FC values within these three subcortical structures and the effect sizes of differences as compared to HC are shown in Figure 2. Both SZ and BD had greater intra-thalamic deficit compared with HC, whereas the MDD group displayed only mild, nonsignificant thalamic alterations indistinguishable from other groups (Figure 2). Although BD and MDD groups showed lower average intra-striatal connectivity, these differences were not statistically significant relative to controls (Figure 2). The MDD group showed significant and the largest effect size for intra-limbic connectivity deficits relative to HC (Figure 1 and Table 2). The SZ and BD groups showed only mild reduced effect sizes of average FC within the limbic region (Figure 2).

### FC difference between subcortical structures

Patients with SZ exhibited significant hyperconnectivity relative to controls among the thalamus, striatum, and limbic regions. Specifically, wide-spread increases in FC were observed between the striatum and thalamus, the striatum and amygdala, and the thalamus and amygdala. In contrast, patients with BD demonstrated reduced FC relative to controls between the thalamus and striatum, as well as between the striatum and limbic structures. Notably, this included significant hypoconnectivity between the amygdala and putamen (peak *t* = −4.17, *p* < 0.0001). Similarly, patients with MDD showed decreased FC relative to controls between the striatum and limbic structures, primarily driven by hypoconnectivity between the amygdala and putamen (peak *t* = −4.4, *p* < 0.00001) (Table 2). In summary, SZ was characterized by hyperconnectivity between the thalamus, striatum, and limbic structures. Hypoconnectivity between the striatum and limbic regions was a shared feature of BD and MDD.

Additionally, we quantified the number of dysconnected region pairs compared to HC, as shown in Table 2. Across the entire analysis, 1,431 pairwise comparisons were conducted. Relative to HC, abnormal FC was observed in 17% of connections in the SZ group, 7.1% in the BD group, and 1.5% in the MDD group (Table 2). We would like to clarify direct disorder-specific comparisons to identify alterations unique to each patient group (SZ versus BD versus MDD). These analyses were performed using post-hoc contrasts within our general linear model framework, specifically targeting the intra- and inter-subcortical connections that showed significant changes compared to HCs. The results of these direct group-by-group contrasts are detailed in the supplementary materials (specifically Supplementary Figure S2) and reveal critical information regarding the hierarchy of subcortical dysconnectivity across the three disorders.

### Two key supplementary analyses intra-subcortical abnormalities

The sensitivity analysis, presented in Supplementary Table 2, highlighted important differences attributed to the effects of medication. Our results demonstrated that, while the inter-subcortical connections showed greater variability and susceptibility to antipsychotic medication status, the core intra-subcortical abnormalities were remarkably stable and persisted even in the antipsychotic-non-use subsample.

We performed an exploratory analysis correlating our primary intra-subcortical connectivity measures with clinical severity across the relevant patient cohorts (Supplementary Figure S3). In SZ, exhibited significant positive correlation between dysconnected pairs and clinical severity with intra-striatal (*r* = 0.14, *p* = 0.045) and intra-thalamic FC (*r* = 0.17, *p* = 0.015), indicating that higher functional integration within the striatum is directly linked to greater overall psychotic symptom severity. In contrast, patients of psychotic symptom severity with BD a significant negative correlation of intra-striatal FC (*r* = −0.24, *p* = 0.006) and intra-thalamic FC (*r* = −0.20, *p* = 0.025). In MDD, all shows no significant correlation between intra-subcortical connectivity and PANSS. However, the intra-thalamic FC and intra-limbic FC is associated with the depressive symptom severity (*r* = 0.22, *p* = 0.007; *r* = 0.20, *p* = 0.011, relatively) in MDD.

## Discussion

Our study compared subcortical resting-state FC across SZ, BD, and MDD. Using a large single-site sample and a high-resolution subcortical parcellation (Tian et al., 2020), we identified both overlapping and distinct connectivity abnormalities across these disorders. SZ was characterized by intra-thalamic hypoconnectivity coupled with intra-striatal hyperconnectivity, whereas BD similarly exhibited intra-thalamic hypoconnectivity but showed reduced intra-striatal connectivity. In contrast, MDD displayed relatively focal hypoconnectivity within limbic structures (notably the amygdala and hippocampus) and only limited intra-thalamic hypoconnectivity. Distinct patterns of FC abnormalities between these three subcortical structures were also demonstrated. These findings underscore both shared and disorder-specific subcortical network disruptions – thalamic connectivity deficits emerge as a common thread in SZ and BD, whereas striatal and limbic circuit alterations differentiate the disorders.

We observed significantly reduced FC within the thalamus SZ, BD, and MDD, indicating a shared intra-thalamic hypoconnectivity. This finding aligns with prior evidence of thalamic dysconnectivity in serious mental illness and is consistent with impaired thalamic gating in psychotic conditions (Ferri et al., 2018; McCormick & Bal, 1994). As a critical relay hub, the thalamus’ reduced internal coherence may contribute to cognitive and sensory-processing deficits in SZ. Longer illness duration may also relate to reduced intra-thalamic connectivity in SZ (Gong et al., 2019). The shared thalamic hypoconnectivity in the three psychiatric disorders suggests overlapping neural deficits that may contribute to common perceptual and cognitive impairments (Anticevic et al., 2014).

Intra-striatal connectivity alterations in SZ and BD were marked but in opposite directions. Individuals with SZ demonstrated **hyperconnectivity** within striatal regions, reflecting excessive synchronous activity in basal ganglia circuits. SZ severity is also linked to increased intra-striatal activity. This was probably not related to the traditional hyperdopaminergic hypothesis in SZ but to a condition known as medication-induced supersensitivity (Chouinard et al., 2017). Previous research studies have shown that striatal connectivity with other brain regions in SZ can predict treatment response (Han et al., 2020; Li et al., 2020; Sarpal et al., 2016). However, only one study to date has reported baseline intra-striatal hyperconnectivity in SZ patients (Li et al., 2020). Another study observed increased intra-striatal activity – measured by fALFF and regional homogeneity – in both treatment-naive patients and after an 8-week course of risperidone monotherapy (Hu et al., 2016). Antipsychotic medication has also been shown to enhance subcortical-frontoparietal connectivity (Blazer et al., 2022), and in some cases, post-treatment connectivity may exceed that of HC, as observed in studies using hippocampal seed regions (Kraguljac et al., 2016). Whether these findings reflect trait markers of SZ, medication-induced changes over time, or are influenced by illness duration or clinical subtypes warrants further investigation. By contrast, BD exhibited reduced intra-striatal connectivity. BD has been shown to have elevated striatal signaling during reward-processing tasks in manic or hypomanic states (Whitton, Treadway, & Pizzagalli, 2015). Few studies have investigated intra-striatum connectivity in BD so far (Okanda Nyatega, Qiang, Jajere Adamu, & Bello Kawuwa, 2022) and most studies showed mixed results in striatum connectivity with other structures (Bi, Che, & Bai, 2022; Vargas et al., 2013; Zhang et al., 2022).

MDD exhibited distinct limbic connectivity deficits, in contrast to relatively intact limbic networks in SZ and BD. Specifically, depressed patients showed reduced FC between the amygdala and hippocampus – key structures for emotion, learning, and memory – consistent with the affective symptoms and memory disturbances of MDD (Barch et al., 2016; Cullen et al., 2014). Given that the amygdala and hippocampus are central to emotion regulation and memory processing, this weakened coupling may underlie the mood dysregulation and negative cognitive bias observed in depression (Zheng et al., 2017). Thus, limbic circuit dysfunction appears relatively specific to MDD, aligning with the prominent sustained negative affect in depression and distinguishing it neurobiologically from SZ and BD. Mood disorder (BD/MDD) severity is often linked to increased integration in the thalamic and limbic systems, while also showing patterns of decreased striatal integration correlating with depressive states, suggesting a complex push–pull of hyper- and hypo-connectivity depending on the subregion and symptom type.

SZ patients exhibited wide-spread hyperconnectivity between subcortical structures, with increased FC across numerous region pairs compared to HC, suggesting a diffuse subcortical network over-engagement, as opposed to poorer fronto-parietal connectivity in SZ (Voineskos et al., 2024). This may be the result of compensatory loops in response to wide-spread neurodevelopmental abnormality in cortical area such as the fronto-temporal and associated cortex (Gallucci et al., 2022; Gratton et al., 2018). By contrast, BD showed a more selective and distinct hypoconnectivity between subcortical regions: striatal–limbic loops, reflecting impairment of mood regulatory circuits in BD (Cotovio & Oliveira-Maia, 2022). Supporting this, other studies have reported abnormally increased neural activity in response to both positive cues and negative cues in BD and MDD (Cotovio & Oliveira-Maia, 2022). Meanwhile, striatum–amygdala hypoconnectivity in BD and MDD may reflect shared deficits in emotional processing (Korgaonkar et al., 2019). MDD had minimal inter-nuclear connectivity changes, reflecting largely preserved subcortical network integrity in depression. This is consistent with clinical observations that individuals with MDD tend to have more controlled symptoms, preserved reality testing, minimal psychosis, and near normal performance on cognitive tasks (Semkovska et al., 2019). These findings suggest a gradient of subcortical network disruption severity, with SZ showing the most extensive dysconnectivity, BD an intermediate pattern, and MDD the least (Tu et al., 2020). While our analysis identified significant alterations in connectivity within and between subcortical structures, we recognized that the functional implications of these isolated changes remain complex, as subcortical regions are integral nodes within larger thalamocortical and corticostriatal networks. However, because subcortical nuclei are topographically organized to correspond with specific cortical territories (Bell & Shine, 2016; Hwang et al., 2017; Ji et al., 2019), these intrinsic connectivity changes serve as high-sensitivity indicators of broader network-wide dysfunction. This focused ROI-based approach provided the statistical power necessary to detect subtle changes in small, deep-brain structures that are frequently obscured by the stringent multiple-comparison corrections required in whole-brain analyses. Consequently, while these findings should be interpreted with caution within the broader neurobiological landscape, they offer a uniquely granular view of subcortical disruptions that may serve as primary drivers of the pathology observed in this cohort.

Our study’s notable strengths – including the large single-site sample and high-resolution subcortical parcellation – enhance confidence in the reliability by standardizing preprocessing pipelines and analytic methods (Botvinik-Nezer et al., 2020; Bowring, Maumet, & Nichols, 2019). However, further validation in other independent and diverse cohorts is warranted to assess the robustness and clinical utility of these connectivity-based markers. Additionally, as this study is cross sectional, we cannot determine causality – that is, whether the observed connectivity differences are preexisting trait markers or consequences of illness and evolving with the course of illness (Parkes, Satterthwaite, & Bassett, 2020; Woo, Chang, Lindquist, & Wager, 2017). Despite employing demographic variables (age, sex, and education level) as covariates of no interest within our general linear model to statistically mitigate their influence, the demographic heterogeneity of the MDD cohort (older age, higher proportion of females, and shorter illness duration) remains a limitation, although this heterogeneity partially reflects the known clinical and prevalence characteristics of MDD. We acknowledge that the specific connectivity differences observed in the MDD cohort may reflect a unique combination of illness-state effects and demographic influence that could not be fully separated through statistical modeling alone. Future large-scale, multisite studies with prospectively matched patient and control groups are necessary to definitively disentangle these effects. Second, most patients were taking psychotropic medications, which may have influenced connectivity patterns despite our attempts to control for medication effects (Chopra et al., 2021). Third, we tried to examine correlations between connectivity measures and clinical variables (e.g. symptom severity), suggesting that a global reduction in the thalamus internal filtering and integration capacity is a prominent, yet varying, downstream feature across all three illnesses. Future studies should employ longitudinal designs, include unmedicated or early illness participants, and integrate clinical assessments to address these issues and clarify how subcortical connectivity relates to symptomatology and outcomes.

In conclusion, this study delineates both transdiagnostic and disorder-specific subcortical connectivity disruptions in SZ, BD, and MDD. The convergence of intra-thalamic hypoconnectivity in SZ, BD, and MDD, alongside divergent striatal and limbic patterns, highlights a complex neurobiological overlap and distinction among these disorders. To our knowledge, this is the first study to compare subcortical connectivity across SZ, BD, and MDD. These findings advance our understanding of subcortical circuit pathology and may inform improved diagnostic differentiation and targeted treatment strategies for these major psychiatric illnesses.

## Supporting information

10.1017/S0033291726103377.sm001 Chang et al. supplementary material Chang et al. supplementary material

## Supplementary material

The supplementary material for this article can be found at http://doi.org/10.1017/S0033291726103377.
