# Distinct and common subcortical functional connectivity revealed across three major psychiatric disorders – CORRIGENDUM

**DOI:** 10.1017/S0033291726105236

**Published:** 2026-07-03

**Authors:** Wan-Chen Chang, Duan-Pei Hung, Mu-Hong Chen, Tung-Ping Su, Ya-Mei Bai, You-Yin Chen, Pei-Chi Tu

**Affiliations:** 1 Department of Biomedical Engineering, National Yang Ming Chiao Tung University, Taipei, Taiwan; 2 Department of Medical Research, Taipei Veterans General Hospital, Taipei, Taiwan; 3 Department of Psychiatry, Taipei Veterans General Hospital, Taipei, Taiwan; 4 Department of Psychiatry, Faculty of Medicine, National Yang Ming Chiao Tung University, Taipei, Taiwan; 5 Department of Psychiatry, Cheng Hsin General Hospital, Taipei, Taiwan; 6 Institute of Brain Science, National Yang Ming Chiao Tung University, Taipei, Taiwan; 7 The Ph.D. Program for Neural Regenerative Medicine, College of Medical Science and Technology, Taipei Medical University, Taipei, Taiwan; 8 Institute of Philosophy of Mind and Cognition, National Yang Ming Chiao Tung University, Taipei, Taiwan

The authors regret the inclusion of an error in the above article.

You-Yin Chen was not listed as a corresponding author in the original article. Both Pei-Chi Tu and You-Yin Chen are corresponding authors.

The “contributed equally” designation is also missing to indicate that Duan-Pei Hung contributed equally to this work.

The article has now been corrected.
