# Supplementary material for: Distinct and common subcortical functional connectivity revealed across three major psychiatric disorders
Source: Psychol Med. 2026 Apr 28;56:e109. doi: 10.1017/S0033291726103377 (PMC13125929; doi:10.1017/S0033291726103377)

Supplementary Table S1. Medication details

| **Dx**  **Medication (n)** | **SZ** | **BD** | **MDD** |
| --- | --- | --- | --- |
| antipsychotics | 67 | 38 | 4 |
| antidepressant | 5 | 7 | 39 |
| mood stabilizers | 3 | 26 | 11 |
| antipsychotics + antidepressant | 26 | 14 | 16 |
| antipsychotics + mood stabilizers | 53 | 66 | 4 |
| antidepressant + mood stabilizers | 1 | 15 | 56 |
| antipsychotics + antidepressant + mood stabilizers | 33 | 22 | 19 |

Supplementary Table S2. Sensitivity analysis of intra- and inter-subcortical connectivity: distinguishing stable illness pathology from antipsychotic medication effects.

| **dx** | **SGAs** | **non-SGAs** | **t** | **p** | **q-_FDR_** | **C1** | **C2** | **D1** | **D2** |
| --- | --- | --- | --- | --- | --- | --- | --- | --- | --- |
| SZ | 0.20 ± 0.30 | 0.00 ± 0.20 | 3.587 | 0.00042 | 0.484 | basal ganglia | hippocampal-amygdala | NAc-core-lh | HIP-head-m2-lh |
|  | 0.30 ± 0.20 | 0.30 ± 0.20 | 3.274 | 0.00126 | 0.674 | basal ganglia | basal ganglia | CAU-body-lh | aGP-rh |
|  | 0.30 ± 0.20 | 0.10 ± 0.30 | 3.178 | 0.00172 | 0.730 | thalamus | thalamus | THA-VPl-lh | THA-VPm-rh |
|  | 0.60 ± 0.20 | 0.50 ± 0.30 | 3.154 | 0.00187 | 0.730 | basal ganglia | basal ganglia | PUT-DA-lh | PUT-VA-rh |
|  | 1.10 ± 0.30 | 1.00 ± 0.20 | 3.153 | 0.00187 | 0.730 | thalamus | hippocampal-amygdala | THA-VPl-lh | HIP-head-m2-rh |
|  | 0.50 ± 0.30 | 0.40 ± 0.30 | 3.059 | 0.00253 | 0.818 | basal ganglia | basal ganglia | PUT-DA-lh | aGP-rh |
|  | 0.40 ± 0.30 | 0.30 ± 0.30 | 3.023 | 0.00284 | 0.818 | thalamus | basal ganglia | THA-VPm-lh | PUT-VP-rh |
|  | 0.30 ± 0.20 | 0.20 ± 0.20 | 2.847 | 0.00489 | 0.997 | basal ganglia | basal ganglia | CAU-DA-lh | PUT-VP-lh |
| BD | 0.20 ± 0.20 | 0.10 ± 0.20 | 3.286 | 0.00121 | 0.674 | thalamus | basal ganglia | THA-DP-lh | CAU-tail-lh |
|  | 0.60 ± 0.30 | 0.70 ± 0.30 | -2.869 | 0.00458 | 0.997 | hippocampal-amygdala | hippocampal-amygdala | mAMY-lh | HIP-body-lh |
|  | 0.00 ± 0.20 | 0.10 ± 0.20 | -2.870 | 0.00458 | 0.997 | basal ganglia | basal ganglia | PUT-DA-lh | PUT-DP-rh |
|  | 0.10 ± 0.20 | 0.20 ± 0.20 | -3.022 | 0.00286 | 0.818 | basal ganglia | basal ganglia | CAU-DA-lh | PUT-DP-lh |
|  | 0.30 ± 0.30 | 0.40 ± 0.20 | -3.353 | 0.00097 | 0.674 | hippocampal-amygdala | basal ganglia | HIP-tail-lh | CAU-body-rh |
|  | 0.20 ± 0.30 | 0.30 ± 0.30 | -3.655 | 0.00033 | 0.484 | hippocampal-amygdala | thalamus | HIP-tail-lh | THA-VPm-rh |
|  | 0.10 ± 0.20 | 0.20 ± 0.20 | -3.911 | 0.00013 | 0.484 | basal ganglia | thalamus | CAU-tail-lh | THA-DAm-lh |
| MDD | 0.80 ± 0.20 | 0.90 ± 0.20 | -2.848 | 0.00489 | 0.997 | basal ganglia | thalamus | CAU-body-lh | THA-VAip-rh |
|  | -0.00 ± 0.20 | 0.00 ± 0.20 | -2.997 | 0.00309 | 0.828 | basal ganglia | thalamus | CAU-VA-lh | THA-DP-rh |
|  | 0.20 ± 0.20 | 0.30 ± 0.20 | -3.069 | 0.00246 | 0.818 | basal ganglia | basal ganglia | NAc-shell-lh | PUT-VP-lh |
|  | 0.50 ± 0.20 | 0.60 ± 0.20 | -3.313 | 0.00111 | 0.674 | basal ganglia | hippocampal-amygdala | NAc-shell-lh | mAMY-lh |
|  | 0.20 ± 0.20 | 0.30 ± 0.20 | -3.571 | 0.00045 | 0.484 | basal ganglia | basal ganglia | NAc-core-lh | PUT-VA-lh |

SGAs: Second-generation antipsychotics; C: complex; D: division

Supplementary Figure S1 Subcortical regions of interest (ROIs) defining the functional connectivity analysis nodes.


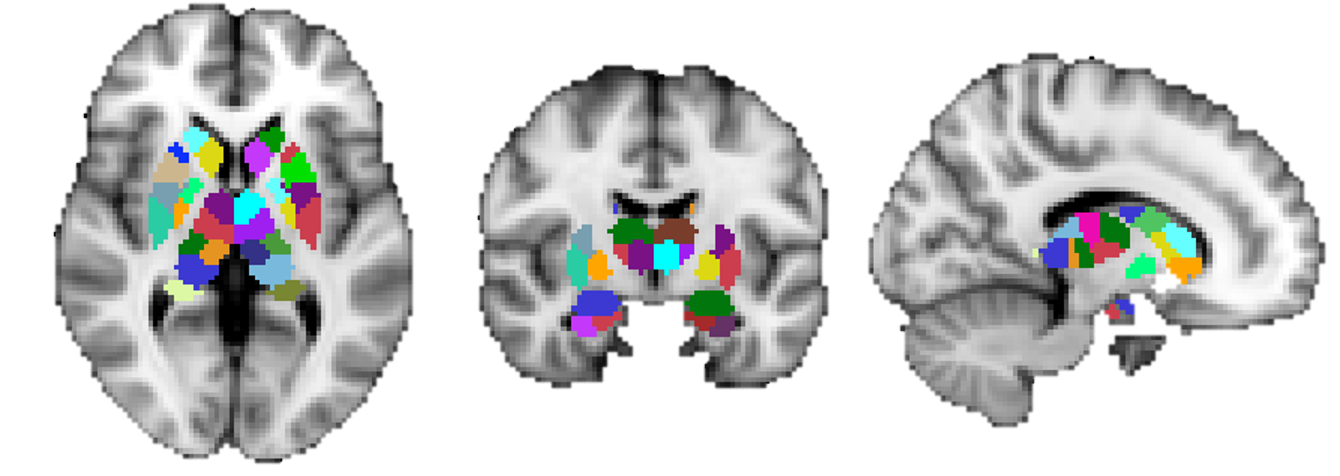


Supplementary Figure S2 Post-hoc comparisons of thalamic, striatal, and limbic functional connectivity in patient groups.


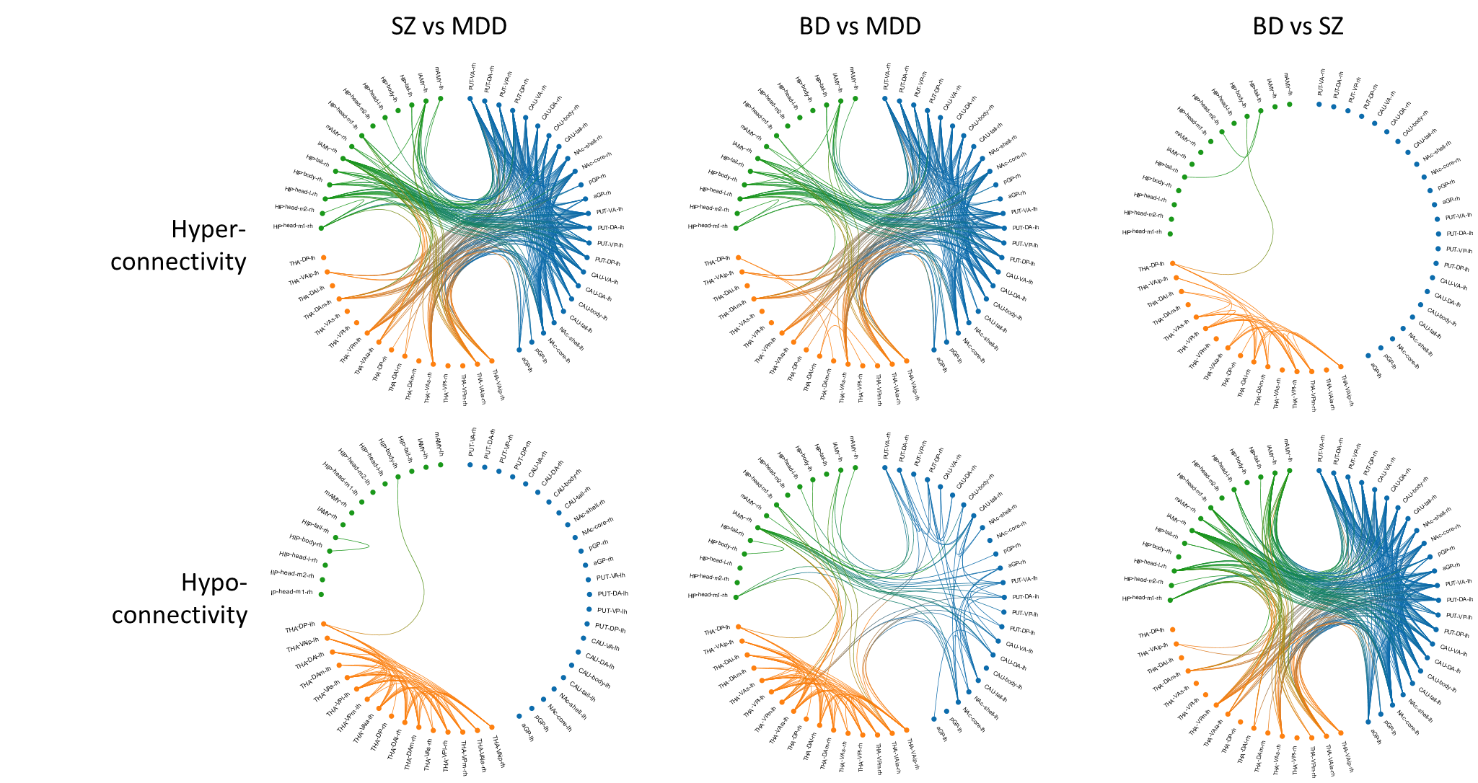


Supplementary Figure S3 Disorder-specific correlation of intra-subcortical functional connectivity with clinical psychotic symptoms.


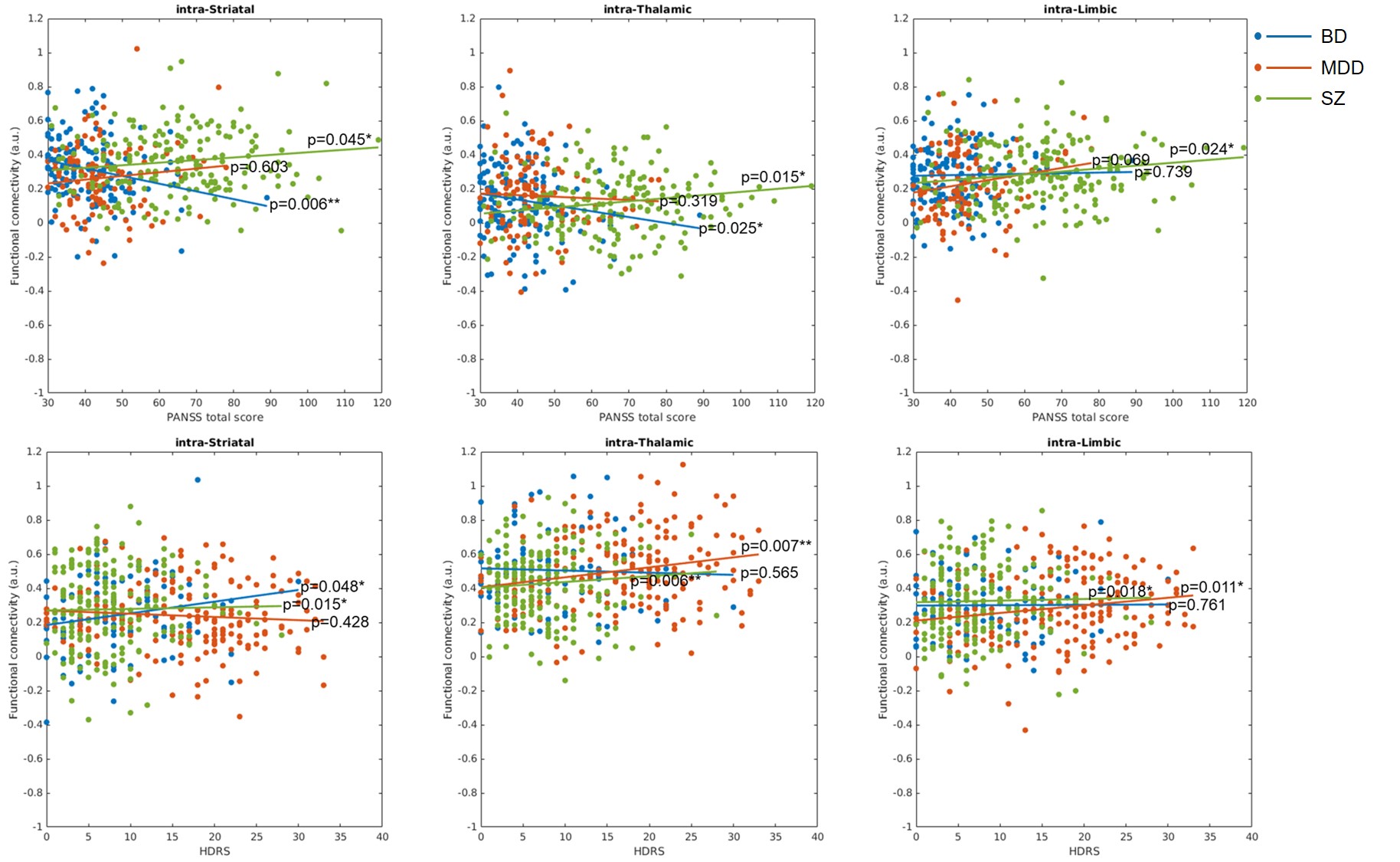

Supplement: Chang et al. supplementary material [file S0033291726103377sup001.docx]
